# Improving Color Accuracy of Colorimetric Sensors

**DOI:** 10.3390/s18041252

**Published:** 2018-04-18

**Authors:** Eric Kirchner, Pim Koeckhoven, Keshav Sivakumar

**Affiliations:** 1 Color Research Group, AkzoNobel Paints & Coatings, 2170 BA Sassenheim, The Netherlands; Pim.Koeckhoven@akzonobel.com; 2 Color Research Group, AkzoNobel Paints & Coatings, 562 114 Bengaluru, India; Keshav.Sivakumar@akzonobel.com

**Keywords:** colorimetric sensor, measurement accuracy

## Abstract

Accurate measurements of reflectance and color require spectrophotometers with prices often exceeding $3000. Recently, new “color instruments” became available with much lower prices, thanks to the availability of inexpensive colorimetric sensors. We investigated the Node+ChromaPro and the Color Muse, launched in 2015 and 2016 by Variable Inc. Both instruments are colorimeters, combining a colorimetric sensor with LED lighting. We investigated color accuracy compared to a high-end spectrophotometer from BYK Gardner. With different sets of samples we find for the Node an average value of dE_CMC_ (1:1) = 1.50, and a maximum of 7.86, when comparing with the 45° geometry of the spectrophotometer. Utilizing measurement data on the Spectral Power Distributions of the LEDs, we developed three methods to improve color accuracy as compared to the spectrophotometer data. We used these methods on different sets of samples with various degrees of gloss, both for training the models underlying the methods and for independent tests of model accuracy. Average color accuracy of the Node+ChromaPro improves from dE_CMC_ (1:1) = 1.82 to 1.16 with respect to spectrophotometer data. The percentage of samples with dE_CMC_ (1:1) < 1.0 increases from 30.9% (uncorrected) to 64%. With the improved color accuracy, these sensors become useful for many more applications.

## 1. Introduction

Accurate measurements of colorimetric properties of reflective materials require conventional spectrophotometers. Their price, typically exceeding $3000 (and often even exceeding $20,000), is beyond the budgets of many small businesses and almost all prosumers. Recently, several new “color instruments” have become available, with prices below $300. To a large extent, this development is the result of inexpensive colorimetric sensors becoming available as a result of market developments in digital cameras and smartphones. The availability of inexpensive color instruments may open up a whole range of markets in which the dramatic drop in price outbalances the inevitable decrease in color accuracy.

A method for improving the accuracy of spectrophotometer measurements by aligning it to a standard is discussed by Berns and Petersen [1]. This method is specific to spectrophotometers and does not apply to readings from a colorimetric sensor, which measures only color coordinates and not the entire spectral reflectance. In the past, colorimeters were used primarily to measure output from light sources and display devices like Cathode Ray Tube displays. These colorimeters do not have their own light source. Techniques to improve the performance of these colorimeters are discussed in [2,3,4]. Most of these techniques are based on matrix transformations of three color signals. However, these methods are not applicable to the low-cost color measuring devices available in the market today, like the Color Muse, which have their own light source.

A different approach originates from processing digital images. Digital cameras have become very common and affordable in the form of smartphone cameras. Some work has been done to use the RGB-based camera images to measure the color of an object. One of the major drawbacks of using a digital camera is the dependence on ambient light for measurement which results in a different color measurement of the same color when measured in different light settings, e.g., under daylight and under fluorescent light. Some methods for improving the color accuracy of digital camera color measurements are given in [5,6,7]. However, the color accuracy obtained by using the CCD of a digital camera is very low, even after correction techniques are applied.

**Figure 1:**
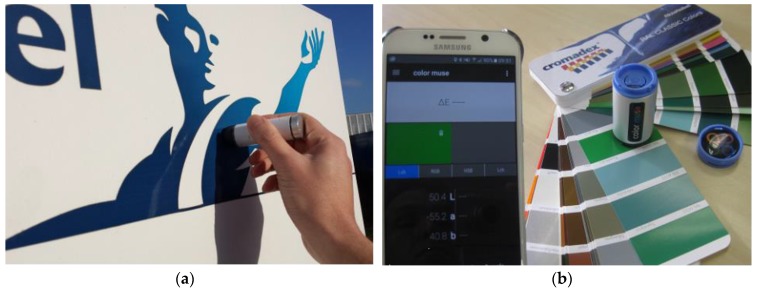
Two low-cost color measurement devices: (**a**) Node+ChromaPro; (**b**) Color Muse.

We investigated two of these recent low-cost color instruments as shown in Figure 1, both produced by Variable Inc. (Chattanooga, TN, USA) [8]. The Node+ChromaPro was launched in 2015 [9,10] as part of a multi-sensor system [11], and is also available as Colour PIN from NCS [12]. The Color Muse became available in 2016 [13]. Both instruments are, in fact, colorimeters, combining a colorimetric sensor with light from 3 to 4 different LEDs. In the case of the Node+ChromaPro, the colorimetric sensor consists of an organic film with red, green and blue filters and an array of photodiodes [14]. The RGB readings can then be converted to CIE Tristimulus (X, Y, Z) data by mathematical conversion, after calibration on a series of test samples [15]. In the case of the Color Muse, the colorimetric sensor uses an interference filter that is designed such that its transmission characteristics closely follow the tristimulus curves of the CIE standard observer [16]. The sensor in the Color Muse is a tristimulus sensor, recently developed by the Austrian company AMS, which is a main supplier of sensors for cell phone manufacturers like Samsung and Apple.

Obviously, the instruments investigated here do not provide full spectral reflection data. We investigated their color accuracy when compared to a high-end spectrophotometer, the multi-angle BYK-mac from BYK Gardner [17]. We also show how conversion methods can be derived that improve the color accuracy of the colorimetric sensors. Similar conversion methods are also expected to improve the color accuracy of other types of colorimetric sensors. With the accuracy improvement method developed in this work, the colorimetric sensors may become useful in many more application areas.

## 2. Materials and Methods

### 2.1. Spectral Power Distributions

**Figure 2:**
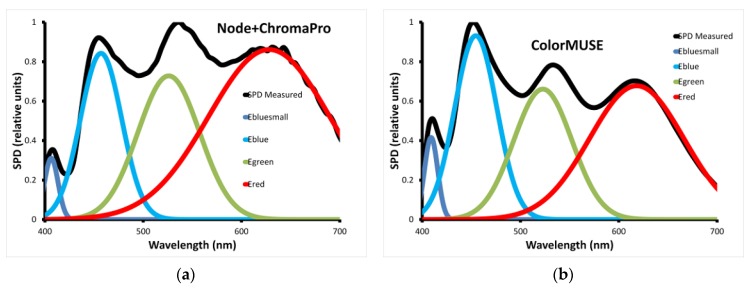
Spectral Power Distribution as measured for (**a**) Node+ChromaPro and (**b**) Color Muse. In both cases, best fit functions are shown for underlying LEDs.

The light source inside the instruments consists of LEDs. During a measurement, red, green and blue LEDs of the Node+ChromaPro are switched on, so the corresponding Spectral Power Distributions (SPDs) are relevant for understanding (and possibly improving) the performance of the instrument. The combined SPD was measured with a CL-500A illuminance spectrophotometer from Konica-Minolta (Tokyo, Japan) as shown in Figure 2. The measured SPD clearly shows contributions from separate LEDs that peak at 409 and 454 (blue), 523 (green) and 618 nm (red). We fitted the combined SPD to a linear combination of three Gaussian curves that we will denote as E_red_(λ), E_green_(λ) and E_blue_(λ) here, with E being an abbreviation for Emission spectrum.

For the Color Muse, the LED lighting is combined into a so-called white LED, but the SPD shows it also consists of red, green and blue LEDs as shown in Figure 2.

### 2.2. Color Accuracy Compared to Spectrophotometer

The low-cost color measurement devices investigated here are no spectrophotometers. As a quantitative measure for their color accuracy, we will compare the CIELAB readings from the Node+ChromaPro and Color Muse with those from a high-end multi-angle spectrophotometer, the BYK-mac from BYK Gardner [17]. Since the measurement geometry of both low-cost instruments is close to the 45/0 geometry, we will use the 45° aspecular angle geometry from the BYK-mac in this comparison. The difference in CIELAB values is expressed here as a color difference dE_CMC_ (1:1), calculated for a 2 degree observer.

We used several sets of test colors in our investigation: (a) the BCRA tiles [18] are high-gloss ceramic tiles that are routinely used for calibration of spectrophotometers. This makes the BCRA tile set relevant for this investigation, although the number of samples is relatively small for training the models, (b) RAL Classic is a well-known set of high-gloss solid colors developed by the German Institute for Quality Assurance and Labeling (RAL) [19], and (c) is a set called RAL Design by the same institute, which is semi-gloss.

Table 1 shows that the average color accuracy of the Node+ChromaPro varies from dE_CMC_ (1:1) = 1.16 to 2.23. High-gloss samples seem to show lower color accuracy according to this table. For the Color Muse, Table 2 shows that the average color accuracy varies from dE_CMC_ (1:1) = 1.68 to 2.57. As observed from Table 1, high-gloss samples show lower color accuracy compared to matte samples.

### 2.3. Methods for Improving Color Accuracy

We investigated the following methods for improving the color accuracy of the Node+ChromaPro and Color Muse:

Method A. We utilize the combined Spectral Power Distribution of the instrument (Figure 2a), and assume that inside the instrument, the colorimetric sensor actually consists of three different sensors with different wavelength sensitivities that we will denote as D_red_ (λ), D_green_ (λ) and D_blue_ (λ), here, with D being an abbreviation for Detector sensitivity spectrum. The three detector sensitivity functions are not specified by the manufacturer, so these are optimized. For every sample that is measured, the three detector sensitivity functions are integrated against the known spectral reflectance curves and the known combined Spectral Power Distribution, resulting in three internal parameters R_int_, G_int_ and B_int_. With a transformation matrix, these are first converted into tristimulus values X, Y and Z, and thence into CIELAB values L’, a’ and b’. Finally, we apply a linear model for each of the CIELAB values, by taking, for example, L_Node_ = c_1_ + c_2_L’. When working with 20 nm spectral resolution, this method involves fitting 63 parameter values: 3 × 16 parameters for the 16 wavelength bands of the three detector sensitivity functions, 9 coefficients for the transformation matrix and 6 coefficients for the final linear transform.

Method B. This method is similar to Method A, but it utilizes the three fitted Spectral Power Distributions of the LEDs inside the instrument (Figure 2a). In Method B, we assume that inside the instrument, only one (type of) colorimetric sensor is used with a wavelength sensitivity denoted as D(λ). The detector sensitivity function is optimized by a fit procedure. For every sample that is measured, the unknown detector sensitivity function is integrated against the known spectral reflectance curves and the three known Spectral Power Distributions, resulting in three internal parameters R_int_, G_int_ and B_int_. Similar to Method A, a transformation matrix and a linear transformation are then applied. For 20 nm spectral data, Method B involves fitting 31 parameter values: 16 parameters for the wavelength-dependent detector sensitivity function, 9 coefficients for the transformation matrix and 6 coefficients for the final linear transform.

Method C. In this method, Reflectance values from the BYK-mac at 45 degrees are used to calculate tristimulus values X_BM_, Y_BM_ and Z_BM_. In the next step, these are correlated to CIELAB values measured by the Node+ChromaPro by assuming linear terms in the tristimulus values and their cubic roots (obviously, this is inspired by the definitions of CIELAB space). Therefore, we use:

L_Node_ = c_0_ + c_1_(Y_BM_)^1/3^ + c_2_Y_BM_
a_Node_ = d_0_ + d_1_(X_BM_)^1/3^ + d_2_X_BM_ + d_3_(Y_BM_)^1/3^ + d_4_Y_BM_
b_Node_ = e_0_ + e_1_(Z_BM_)^1/3^ + e_2_Z_BM_ + e_3_(Z_BM_)^1/3^ + e_4_Y_BM_

This method involves fitting 13 parameter values.

## 3. Results and Discussion

### 3.1. Results for the Node+ChromaPro

We found that the performance in color accuracy varies depending on the sets of samples used for optimizing the parameter values. Best results were obtained when using the RAL Design set for training the model and using Method B for improving the color accuracy. The average color accuracy of the Node+ChromaPro is found to improve from dE_CMC_ = 1.82 to 1.16 with respect to BYK-mac data, as tested on an independent set of samples.

### 3.2. Results for the Color Muse

The improvement methods that were developed for the Node+ChromaPro were also applied to the Color Muse. For the Color Muse, we found the best color accuracy when Method A was used for the correction, and if a separate set of 46 high-gloss solid colored samples was used for training. In that case, the average color accuracy of the Color Muse improves from dE_CMC_ (1:1) = 1.92 to 1.17 with respect to BYK-mac data. For Method C, the average color difference does not reach below dE_CMC_ (1:1) = 1.32. Therefore, we find that the best performing improvement model differs for the Node+ChromaPro and Color Muse.

Table 3 shows that for the Color Muse, a substantial improvement in color accuracy is found.

For many practical implementations, an instrument with an accuracy within dE_CMC_ (1:1) < 1.0 or dE_CMC_ (1:1) < 1.5 makes it useful, since these are approximately the accuracies needed for visual color matches. Table 3 shows that for the Color Muse and the Node, the improvement method proposed in this article increases the percentage of satisfactory color matches by 30 to 40 percent. Instead of achieving the required accuracy for 23.5–52.6 percent, after applying the improvement method this increases to 58.2–82.2 percent. This makes these colorimetric sensors much more useful for practical applications.

## 4. Conclusions

We have investigated the color accuracy of the colorimetric sensors that are part of two color measurement devices, the Node+ChromaPro and the Color Muse. We found that compared to spectrophotometer data, these instruments result in average color accuracy of dE_CMC_ (1:1) = 1.82 and 1.92 for the Node+ChromaPro and the Color Muse, respectively. With the improvement methods proposed in this article, the accuracy of these colorimetric sensors can be improved to dE_CMC_ (1:1) = 1.16 and 1.17 for Node+ChromaPro and Color Muse. The improvement methods are defined in a generic way that is expected to also lead to improvements in color accuracy of other types of colorimetric sensors.

## Figures and Tables

**Table 1:** Color accuracy, expressed as dE_CMC_ (1:1), of Node+ChromaPro with respect to BYK-mac in the 45° geometry, for different sets of test colors.

Color Difference	BCRA Tiles	RAL Classic	RAL Design
Average	2.23	1.84	1.16
Maximum	8.05	7.40	4.66
Standard deviation	2.00	1.18	0.66
Number of samples	39	167	199

**Table 2:** Color accuracy, expressed as dE_CMC_ (1:1), of ColorMUSE with respect to BYK-mac in the 45° geometry, for different sets of test colors.

Color difference	BCRA Tiles	EIA Panels	RAL Design
Average	2.57	2.12	1.68
Maximum	10.67	10.33	4.64
Standard deviation	2.53	1.57	0.75
Number of samples	26	46	199

**Table 3:** Color accuracy, expressed as percentages of samples with dE_CMC_ (1:1) smaller than specified values.

Color Accuracy	Node Uncorrected	Node Method B	Color Muse Uncorrected	Color Muse Method A
dE_CMC_ (1:1) < 0.5	5.4%	21.9%	7.5%	22.5%
dE_CMC_ (1:1) < 1.0	28.3%	58.2%	23.5%	62.5%
dE_CMC_ (1:1) < 1.5	51.5%	79.4%	52.6%	82.2%
dE_CMC_ (1:1) < 2.0	71.1%	86.5%	76.4%	90.8%
dE_CMC_ (1:1) < 3.0	88.0%	93.3%	88.2%	93.4%
dE_CMC_ (1:1) < 5.0	95.4%	96.8%	93.4%	96.7%
dE_CMC_ (1:1) < 10.0	99.4%	99.8%	100%	100%
